# Phosphorylation of NFATC1 at PIM1 target sites is essential for its ability to promote prostate cancer cell migration and invasion

**DOI:** 10.1186/s12964-019-0463-y

**Published:** 2019-11-15

**Authors:** Sini K. Eerola, Niina M. Santio, Sanni Rinne, Petri Kouvonen, Garry L. Corthals, Mauro Scaravilli, Giovanni Scala, Angela Serra, Dario Greco, Pekka Ruusuvuori, Leena Latonen, Eeva-Marja Rainio, Tapio Visakorpi, Päivi J. Koskinen

**Affiliations:** 1 Department of Biology, University of Turku, Vesilinnantie 5, FI-20500 Turku, Finland; 2 Faculty of Medicine and Health Technology, Tampere University and Tays Cancer Center, Tampere University Hospital, Tampere, Finland; 3 Turku Centre for Biotechnology, University of Turku, Turku, Finland; 4 Institute of Biomedicine, University of Eastern Finland, Kuopio, Finland; 5 University of Helsinki, Helsinki, Finland; 6 Signal processing laboratory, Tampere University of Technology, Pori, Finland; 7 Fimlab Laboratories, Tampere, Finland

**Keywords:** Prostate cancer, Metastatic carcinoma, NFATC1, PIM kinases, Cell motility

## Abstract

**Background:**

Progression of prostate cancer from benign local tumors to metastatic carcinomas is a multistep process. Here we have investigated the signaling pathways that support migration and invasion of prostate cancer cells, focusing on the role of the NFATC1 transcription factor and its post-translational modifications. We have previously identified NFATC1 as a substrate for the PIM1 kinase and shown that PIM1-dependent phosphorylation increases NFATC1 activity without affecting its subcellular localization. Both PIM kinases and NFATC1 have been reported to promote cancer cell migration, invasion and angiogenesis, but it has remained unclear whether the effects of NFATC1 are phosphorylation-dependent and which downstream targets are involved.

**Methods:**

We used mass spectrometry to identify PIM1 phosphorylation target sites in NFATC1, and analysed their functional roles in three prostate cancer cell lines by comparing phosphodeficient mutants to wild-type NFATC1. We used luciferase assays to determine effects of phosphorylation on NFAT-dependent transcriptional activity, and migration and invasion assays to evaluate effects on cell motility. We also performed a microarray analysis to identify novel PIM1/NFATC1 targets, and validated one of them with both cellular expression analyses and in silico in clinical prostate cancer data sets.

**Results:**

Here we have identified ten PIM1 target sites in NFATC1 and found that prevention of their phosphorylation significantly decreases the transcriptional activity as well as the pro-migratory and pro-invasive effects of NFATC1 in prostate cancer cells. We observed that also PIM2 and PIM3 can phosphorylate NFATC1, and identified several novel putative PIM1/NFATC1 target genes. These include the ITGA5 integrin, which is differentially expressed in the presence of wild-type versus phosphorylation-deficient NFATC1, and which is coexpressed with PIM1 and NFATC1 in clinical prostate cancer specimens.

**Conclusions:**

Based on our data, phosphorylation of PIM1 target sites stimulates NFATC1 activity and enhances its ability to promote prostate cancer cell migration and invasion. Therefore, inhibition of the interplay between PIM kinases and NFATC1 may have therapeutic implications for patients with metastatic forms of cancer.

**Graphical abstract:**

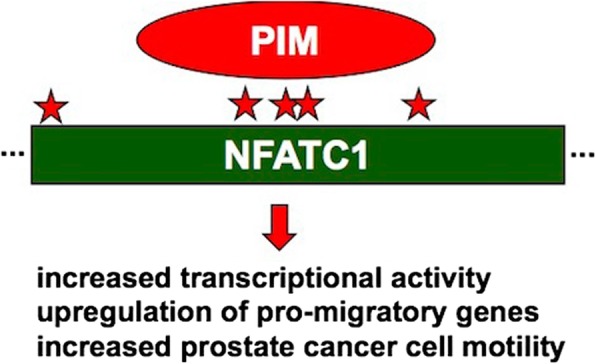

## Background

Prostate cancer is globally one of the most prevalent cancers in men. Locally restricted prostate cancer is usually not fatal, but there is a clear need for effective therapies to prevent or stop progression of local tumors to a metastatic state spreading to bones and other vital organs. Formation of metastases is a multistep process, which includes detachment of cancer cells from the primary tumor, migration, adhesion and invasion of cancer cells into blood or lymph vessels, and infiltration of the cells to secondary sites. Thus, improved understanding of the proteins and signaling pathways that regulate the metastatic growth of cancer cells is essential when developing therapies to treat prostate cancer patients.

NFAT (Nuclear Factor of Activated T cells) transcription factors are ubiquitously expressed in human tissues, where they control cellular processes, such as immune responses [1]. However, one of the family members, NFATC1, has also been shown to act as an oncogene that promotes cancer cell proliferation and transformation [2]. Accordingly, elevated levels as well as increased transcriptional activity of NFATC1 have been detected in both solid cancers and hematological malignancies. NFATC1 has been shown to support cell migration or invasion in multiple types of cancer, such as ovarian, breast and prostate cancer as well as glioblastoma [3–7]. Furthermore, it has been reported to support metastatic behavior of prostate or breast cancer cells via increased osteoclastogenesis [8, 9].

Both the subcellular localization and transcriptional activity of NFAT proteins are post-translationally regulated. Most previously identified phosphorylation sites in NFATC1 have been located to the serine-rich regions (SRRs) and SPXX motifs within the NFAT homology region [10, 11]. Phosphorylation of these sites by kinases such as PKA and GSK3 results in nuclear exit and inactivation of NFATC1. By contrast, dephosphorylation of these sites by the calcium-dependent phosphatase calcineurin leads to nuclear translocation and transcriptional activation.

We have previously shown that the oncogenic PIM1 kinase directly interacts with NFATC1 and phosphorylates it in vitro [12]. However, in contrast to other kinases, PIM1 does not affect the subcellular localization of NFATC1, but stimulates its transcriptional activity in both immune and neuronal cells [12, 13]. PIM1 belongs to a family of three serine/threonine-specific kinases, which have partially overlapping expression patterns, but share several functions to support cell proliferation and survival [14–16]. Increased expression of PIM family members has been detected both in hematological malignancies and in solid tumors. In prostate cancer, overexpression of either PIM1 or PIM3 positively correlates with tumor size, aggressiveness and/or poor patient survival [17–21]. Furthermore, PIM kinases have been linked to regulation of prostate cancer cell motility in several cell-based and animal models, where they have supported cell migration, invasion, tumor angiogenesis and the formation of metastases [4, 16, 22]. As also NFATC1 promotes motility of prostate cancer cells and as PIM-selective inhibitors can block this [4], we now wanted to investigate whether or not PIM-dependent phosphorylation of NFATC1 is important for migration and invasion of prostate cancer cells. Therefore, we identified and mutated the PIM targets sites from NFATC1 and analysed the impact of these mutations in three prostate cancer cell lines, the hormone-insensitive PC-3 and DU-145 cells and the hormone-sensitive LNCaP cells. We also performed a microarray analysis to identify putative phosphorylation-dependent target genes for NFATC1.

## Methods

### Cell culture

The cell culture conditions for prostate epithelial adenocarcinoma cell line PC-3 and the stable cell lines overexpressing human PIM1 have been previously described [22]. DU145 and LNCaP prostate cancer cell lines were obtained from American Type Culture Collection (Manassas, VA) and cultured under recommended conditions. For transient transfections, Fugene 6 or HD reagents (Promega, Fitchburg, WI, USA) were used in 1:2 or 1:3 ratio to DNA according to manufacturer’s instructions. All the cell lines were frequently tested for mycoplasma contamination. Viability of cells was analysed by the MTT assay [4] or the AlamarBlue® cell viability assay (Thermo Fisher Scientific, Waltham, MA, USA).

### DNA constructs and cloning

The pcDNA3.1/V5-HisC, pGEX-6P-1 and pTag-RFP vectors expressing wild-type (WT) or kinase-deficient (KD) human PIM1, 2 or 3 or mouse PIM3 have been previously described [23]. The NFAT-luciferase reporter plasmids as well as wild-type (WT), N-terminally truncated (amino acids 1–418), dominant negative (DN, amino acids 410–680) and constitutively active SRR mutant (mSRR) human NFATC1 expression vectors based on pGEX-3X or pBJ5-Flag were kindly provided by the group of G.R. Crabtree (Stanford University, CA, USA) [10, 24]. Truncated NFATC1 was digested from pGEX-3X with PflMI and ligated to pEYFP-C2 (Clontech Laboratories, Mountain View, CA, USA). Full-length NFATC1 was multiplied by PCR from pBJ5-NFATC1-Flag by using a forward primer (5′ GCG GTA CCG CCA CCA TGG ACT ACA AGG CA 3′) and a reverse primer (5′ CCC GGA TCC CTG CGT CTT TAG 3′), digested with KpnI and BamHI, and ligated into pFlag-CMV-2 (Sigma-Aldrich), from where it was further transfered to pEGFP-C3 (Clontech) by BglI and BamHI digestion, followed by ligation.

### NFATC1 mutagenesis

The QuikChange™ site-directed mutagenesis kit (Stratagene, Agilent Technologies, Santa Clara, CA, USA) was used to prepare phosphodeficient mutants of NFATC1. Mutations to replace serines or threonines with alanine residues were introduced into ten PIM1 target sites with the help of five different primer pairs (Additional file 1: Table S1), resulting in production of double mutant (DM, two primer pairs, 1–2), triple mutant (TM, three primer pairs, 3–5) or multi mutant (MM, all primer pairs) NFATC1.

### In vitro kinase assays

GST fusion proteins were produced in the *E. coli* BL21 strain as previously described [25] with minor modifications. Protein production was induced with 0,5 mM IPTG and protease activity was inhibited by Aprotinin (1:200; Sigma-Aldrich) during cell lysis. Proteins were either eluted as fusion proteins or cleaved by the PreScission protease according to manufacturer’s protocol (GE Healthcare Life Sciences, Little Chalfont, UK). For in vitro kinase assays, cleaved PIM kinase (0.5 μg) and GST-tagged NFATC1 (amino acids 1–418) fusion protein (1 μg) were mixed prior to addition of the 2x kinase buffer (20 mM Pipes, pH 7.0, 5 mM MnCl_2_, 0.25 mM β-glycerophophate, 0.4 mM spermine, 10 μM ATP) with 0.5 MBq of [^32^P] adenosine triphosphate. To inhibit PIM kinase activity, samples were pre-treated for 15 min with 10 μM DHPCC-9, a pan-PIM inhibitor, which was kindly provided by P. Moreau (University of Clermont Auvergne, France) and dissolved in 0,1% DMSO. This ATP-competitive pyrrolocarbazole compound selectively inhibits catalytic activities of all PIM family members in vitro [26], in cell-based assays [4] and in mice xenografted with PIM-expressing prostate cancer cells [22]. After 15 to 30 min kinase reactions at 30 °C, samples were heated in 2x Laemmli sample buffer (LSB) for 5 min at 95 °C. Phosphorylated proteins were resolved in SDS-PAGE, stained by Page Blue solution (Thermo Fisher Scientific) and detected by autoradiography.

### Identification of NFATC1 in vivo phosphorylation sites by mass spectrometry

PC-3 cells were transiently transfected with the pEYFP-NFATC1 expression vector. After 48 h, cells were stimulated with TPA and IM for 1 h prior to cell lysis in RIPA buffer supplemented with complete mini EDTA-free protease inhibitors (Roche, Basel, Switzerland). Protein concentrations were determined by the DC Lowry method (Bio-Rad Laboratories, Inc., Hercules, CA, USA). 1 mg aliquots of proteins were mixed with Chromotek-GFP-Trap® Magnetic beads (Allele Biotechnology, San Diego, CA, USA), after which GFP-tagged proteins were immunoprecipitated according to manufacturer’s protocol, heated in 2x LSB, resolved in 10% Bis-Tris gel (Bio-Rad) and stained with colloidal coomassie blue solution (Thermo Fisher Scientific). NFATC1 protein isolation, trypsin digestion and titanium dioxide enrichment without salt extraction were performed as previously described [27, 28]. Thereafter, samples were analysed by LTQ Orbitrap Velos mass spectrometer (Thermo Fisher Scientific), using the HCD Top 10 method with 10 min gradient and mass value of 300 to 2000.

### Luciferase assays

To measure NFAT-dependent transcriptional activity, PC-3 cells were transiently transfected with the pGL3-IL-2-luciferase reporter and either pBJ5-NFATC1-Flag or an empty control vector. To stimulate NFATC1 activity and nuclear translocation, cells were treated for 7 h with 15 ng/ml of 12–0-tetradecanoyl-phorbol-13-asetate (TPA; Sigma-Aldrich, St. Louis, MO, USA) in DMSO and 1 μM ionomycin (IM; Merck KGaA, Darmstadt, Germany) in EtOH. To inhibit PIM kinase activity, cells were treated for 24 h with 10 μM DHPCC-9 in 0,1% DMSO. As controls for all chemical compounds, their solvents were used. 24 or 48 h after transfections, cells were collected, lysed in 1% NP-40 buffer by repeated freezing and thawing, and analysed for luciferase activity using the Luminoscan Ascent luminometer (Thermo Fisher Scientific).

To compare activities of wild-type (WT) and multi mutant (MM) NFATC1 in PC-3, DU-145 and LNCaP cell lines, cells were transiently transfected with the pGL3-NFAT-luciferase reporter and either WT or MM pCMV-NFATC1-Flag or an empty control vector. Renilla luciferase (pRLTk; Promega) was co-transfected as an internal transfection efficiency control. Part of the cells were treated with TPA and IM and/or DHPCC-9 as described above. To inhibit calcineurin activity and thereby also nuclear translocation of NFATC1, cells were treated for 24 h with 1 μM cyclosporine A (CsA; Merck) in EtOH. Luciferase assays with four parallel samples were performed on 96-well plates using the Dual-Glo® Luciferase Assay System (Promega) according to manufacturer’s protocol. Luciferase activities were measured with the EnVision 2104 Multilabel Reader (Perkin Elmer, Waltham, MA, USA). The results were presented as relative luciferase activity (RLU) corresponding to the firefly luciferase light emission values normalized against renilla luciferase light emission values.

### Localization assays

To determine the subcellular localizations of wild-type and mutant NFATC1 proteins, PC-3 cells plated on coverslips were transiently transfected with Flag-tagged expression vectors. After 48 h, cells were fixed, permeabilized and stained with anti-Flag antibody (Sigma-Aldrich) and Alexa Fluor™ 488 labelled anti-mouse secondary antibody (Thermo Fisher Scientific). Samples were imaged and analysed with the Zeiss ApoTome.2 fluorescence microscope and Zen lite 2012 software. Approximately 15 images were taken from each sample.

### Fluorescence-lifetime imaging method (FLIM)

To visualize interactions between RFP-tagged PIM1 and GFP-tagged NFATC1, PC-3 cells plated on coverslips were transiently transfected with the corresponding expression vectors and/or their empty controls. Part of the samples were treated overnight with DMSO or 10 μM DHPCC-9. 48 h after transfection, cell samples were fixed with 4% PFA and mounted with Mowiol. First, physical interactions between tagged proteins were measured by analysing GFP lifetime with Lambert Instruments Fluorescence Lifetime Attachment (LIFA) and LI-FLIM software as previously described [23]. Then co-localization of proteins was imaged by Zeiss LSM 780 confocal microscope and by sequential scanning with ZEN lite 2012 software. Excitation wavelengths were 488 nm (GFP) and 561 nm (RFP), and emission wavelengths 500–535 nm (GFP) and 599–651 nm (RFP). Image analyses were performed with the ImageJ® software (Wayne Rasband, NIH, USA).

### Wound healing assays

PC-3 or DU-145 cells were transiently transfected with wild-type or mutant NFATC1 expression vectors. 24 h later, samples were treated with either DMSO or 10 μM DHPCC-9. To confirm that changes in cell migration were not due to changes in cell proliferation, 15 μg/ml of the anti-proliferative agent mitomycin C (Sigma-Aldrich) was used. Scratching of the wounds, microscopy and image analyses of PC-3 cells were performed as previously described [4]. Imaging of DU-145 cells was performed with CM Technologies Cell-IQ (D.I. Biotech, Korea) by using 4x objective and image analysis with the Cell-IQ software 4.3 and scratch wound measurement tool.

### Boyden chamber invasion assays

One day after transfection, invasiveness of PC-3 cells was analysed using cell culture invasion inserts of 8 μm pore size (Corning BioCoat™ Matrigel® Invasion Chamber, Bedford, MA, USA) according to manufacturer’s instructions. For this purpose, cells were suspended in DMEM supplemented with 1% BSA (20,000 cells/each chamber) and either DMSO or 10 μM DHPCC-9. Conditioned medium from confluent MG-63 human osteosarcoma cells was used as a chemoattractant [29]. Cells were incubated for 48 h, after which insert membranes were fixed for 2 min in methanol and stained for 10 min with 0,2% crystal violet in methanol. Then they were cut out from the inserts and mounted with immersion oil. Invaded cells on the membranes were scanned by the Olympus BX51 scanner with Surveyor software and analysed by automated image analysis. Results were verified by manual counting with the ImageJ® software from 5 random fields of each membrane.

### Gelatinase activity assay

Gelatinase activity assay was performed with InnoZyme™ gelatinase (MMP-2/MMP-9) fluorogenic activity assay kit (Merck) according to manufacturer’s instructions. Medium samples for the assay were collected from the upper chambers of invasion inserts after the invasion assays described above. Samples were incubated at + 37 °C for 3 h protected from light. Fluorescence was then measured with the Envision plate reader (Perkin Elmer) with an excitation wavelength of 320 nm and an emission wavelength of 405 nm.

### Western blotting

Cells were lysed in 2x LSB and heated at 95 °C for 5 min. Proteins were separated by SDS-PAGE, immobilized onto PVDF-membrane (EDM Millipore, Merck) and incubated overnight with anti-PIM-1 (1:500, 12H8; Santa Cruz Biotechnology, Santa Cruz, CA, USA), anti-PIM-2 (1:1000, D1D2; Cell Signaling Technology, Danvers, MA, USA), anti-PIM-3 (1:1000, D17C9; Cell Signaling Technology), anti-NFATC1 (1:500, Santa Cruz Biotechnology), anti-V5 (1:500, Invitrogen, Carlsbad, CA, USA), anti-Flag (1:500, F1804; Sigma-Aldrich), anti-ACTB (anti-β-actin; 1:1000, 13E5, #4970S, Cell signaling Technology), anti-GAPDH (1:50000, Sigma-Aldrich), anti-β Tubulin (1:40000, Sigma-Aldrich) or anti-Fibrillarin (1:1000, Cell Signaling Technology) antibodies. After incubations with secondary antibodies, chemiluminescence reactions were generated using either Amersham™ ECL Plus or ECL Prime reagents (GE Healthcare).

### Microarray analyses

For microarray analyses, PC-3 cells with or without stable PIM1 overexpression were transiently transfected with wild-type (WT) or multi mutant (MM) NFATC1 expression vectors. At the following day, total RNAs were extracted using the TRIzol reagent (Invitrogen, Carlsbad, CA, USA) according to the manufacturer’s protocol. The samples were then labelled and hybridized using the Agilent whole genome oligo microarray platform on Human Gene Expression v2 4x44K Microarray slides (G4845A; Agilent Technologies, Palo Alto, CA, USA). The slides were scanned on the Agilent C-Scanner and the raw expression values were extracted using the Agilent Feature Extraction software v. 11.0.1.1. Raw mRNA expression values were imported using limma read.maimages function. Low quality probes were filtered using the distribution of negative control probes as a reference. In particular, only probes whose raw expression values were higher than the 90th percentile of negative control probes were retained for successive analysis. Expression values were log2 transformed, quantile normalized between samples and median aggregated at the gene symbol level using Agilent annotation. A limma-based approach [30] was then applied to estimate the difference in average expression in each comparison. A fold-change cutoff (≥0.1) and *p*-value of (< 0.05) were used to determine differential gene expression.

### Canonical pathway analysis

IPA (Ingenuity Pathway Analysis, Ingenuity Systems) was used for functional enrichment and detection of pathways with significant alterations based on microarray gene expressions. In canonical pathway analysis -log(*p*-values) over threshold 2.5 were considered significant.

### Real-time quantitative polymerase chain reaction (qRT-PCR)

*PIM*, *NFATC1* and *ITGA5* expression levels were determined from total RNAs isolated from PC-3 cells as described above. Quantitative real-time PCR was performed using random hexamere primers, Maxima reverse transcriptase (Thermo Scientific), Maxima SYBR Green qPCR Master Mix (Thermo Fischer Scientific) and the CFX96™ Real-Time PCR Detection System (Bio-Rad Laboratories, Inc.). Each sample was run in triplicate, and expression values were normalized against the TATA-binding protein (TBP). Sequences of all primers (Sigma-Aldrich) for qRT-PCR are described in the Additional file 1: Table S2.

### Gene correlation analyses

Three distinct clinical data sets were used to assess correlations between two different genes in clinical prostate cancer patient samples: The Cancer Genome Atlas (TCGA) - Prostate adenocarcinoma RNA-Sequencing data [31], Integrative Genomic Profiling of Human Prostate Cancer microarray data [32] and Tampere PC sequencing data [33].

### Statistical analyses

The statistical significance of data from luciferase, wound healing, FLIM and cell viability assays was determined using the two-sided *t*-test. Cell invasion and gelatinase activity data were analysed by using the unpaired two-sided *t-*test or Wilcoxon matched pairs test. In RT-qPCR data validation, *P*-values were determined by the Mann-Whitney U-test. In gene correlation analyses, Pearson correlation coefficient and *P*-values were determined according to Gaussian populations. In all analyses, a *P*-value < 0.05 was considered statistically significant (*), *P* < 0.01 (**) and *P* < 0.001 (***). Error bars represent standard deviation (SD) values in each graph. Statistical analyses were performed using the GraphPad Prism version 5.02 (GraphPad Software, La Jolla, CA, USA).

## Results

### NFATC1 is endogenously expressed and constitutively active in PC-3 cells

**Fig. 1:**
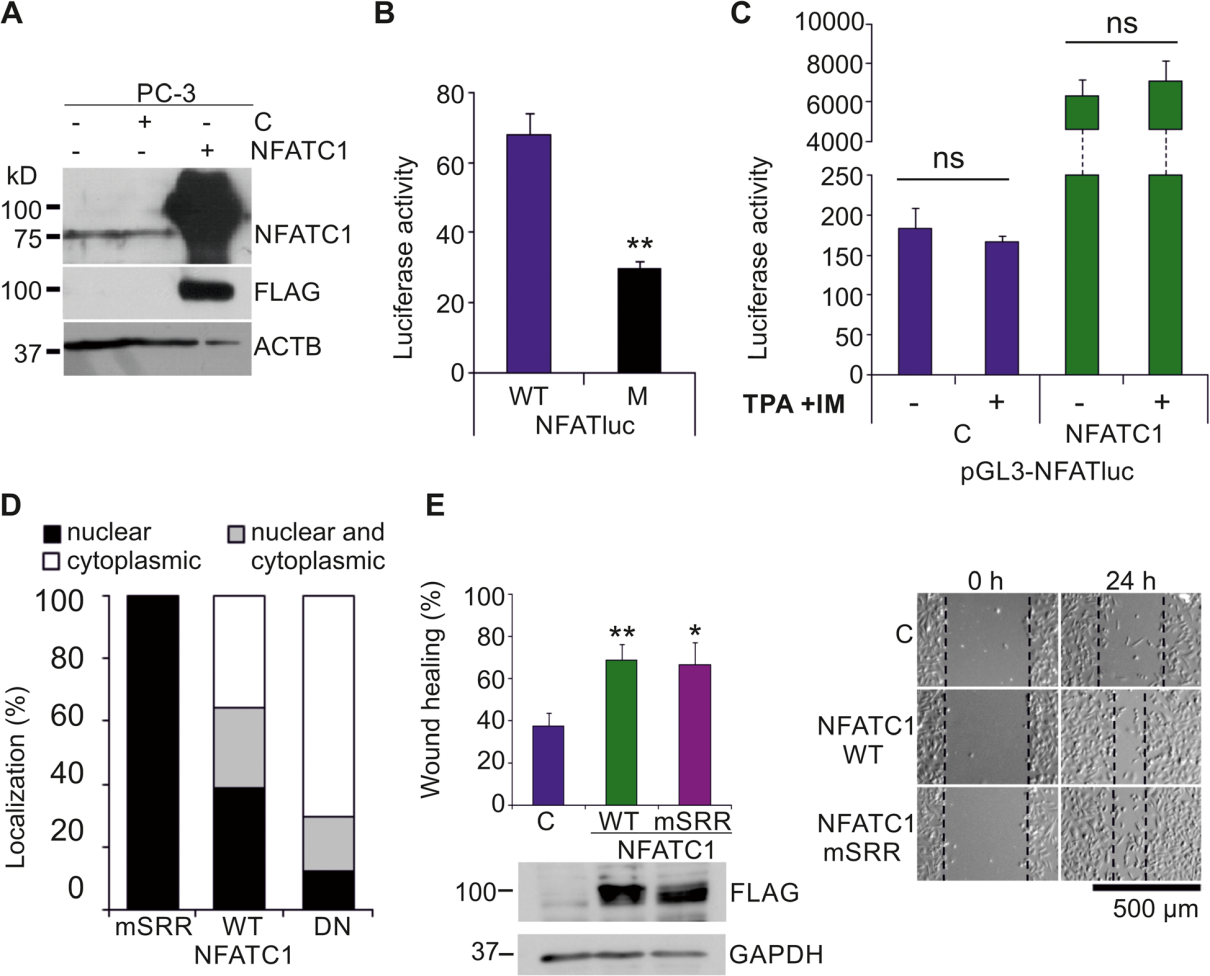
NFATC1 is constitutively active in PC-3 cells. Flag-tagged NFATC1 or its mutated derivatives were transiently expressed in PC-3 prostate cancer cells. Untransfected (−) or mock-transfected cells were used as controls. **a** The endogenous or ectopic expression levels of NFATC1 were analysed by Western blotting with antibodies against NFATc1 or Flag, while ACTB staining was used as a loading control. **b** The endogenous NFAT activity of PC-3 cells was measured by luciferase assays, using transiently transfected reporters with wild-type (WT) or mutated (M) NFAT binding sites. Shown are mean luciferase activities from two independent experiments. **c** The effects of TPA and ionomycin on NFAT activity were measured by luciferase assays. Shown are luciferase activities of duplicate samples from one representative experiment. **d** Subcellular localizations of transiently expressed wild-type (WT) NFATC1, the constitutively active (mSRR) mutant and the dominant negative (DN) mutant were analysed by confocal microscopy after staining with anti-Flag antibody. Shown are average localization patterns from one experiment with three parallel samples. **e** The abilities of WT NFATC1 and the mSRR mutant to promote cell motility were analysed by wound healing assays from three parallel samples. Equivalent expression of these proteins was confirmed by Western blotting with anti-Flag antibody, while GAPDH staining was used as a loading control

As we had previously shown both PIM kinases and NFATC1 to be essential for the motility of PC-3 prostate cancer cells [4], we decided to use these cells in order to investigate in more detail the functional interactions between PIM and NFATC1 proteins. When we analysed the basal expression and transcriptional activity of NFATC1 in PC-3 cells, Western blotting with NFATC1 antibodies detected an endogenously expressed protein with the expected size of approximately 75 kDa (Fig. 1a). NFAT-dependent luciferase assays in turn revealed endogenous NFAT activity, which was dependent on the presence of NFAT binding sites (Fig. 1b), and which was enhanced by ectopic overexpression of NFATC1, but not by stimulation of cells with TPA and the calcium ionophore ionomycin (Fig. 1c). This was surprising, since usually the nuclear translocation and activation of NFATC1 is tightly regulated in a calcium- and calcineurin-dependent fashion [1, 2]. To determine the subcellular localization of NFATC1 in PC-3 cells, we transiently expressed there wild-type (WT) or mutant NFATC1 proteins (24; Table 1). While the dominant negative (DN) mutant was mostly retained in the cytoplasm and the constitutively active (mSRR) mutant in the nucleus, the WT protein could be detected in both compartments (Fig. 1d, Additional file 2: Figure S1A), suggesting that it can shuttle between the compartments of PC-3 cells. When we carried out wound healing assays to compare the effects of WT and mSRR NFATC1 on cell migration, we noticed that both of them enhanced cell motility as compared to control cells (Fig. 1e), while no major changes were observed in cell viability (Additional file 2: Figure S1B).

**Table 1:** Different NFATC1 forms and mutants used in the experiments

NFATC1 proteins	Mutated sites	Length
Wild type (WT)	none	full-length
Dominant negative (DN)	none	410–680 aa
Constitutively active (mSRR)	all 11 serines mutated to alanines in the SRR (172–194)	1–418 aa
Double mutant (DM)	S245, S269	full-length
Triple mutant (TM)	S151, S153, T154, S256, S257, S335, T338, T339	full-length
Multi mutant (MM)	S151, S153, T154, S245, S256, S257, S269, S335, T338, T339	full-length

The amino acid substitutions (from serine or threonine to alanine) and other mutations in NFATC1 and the length of each mutant protein used in this study

### PIM kinases phosphorylate NFATC1 in several serine and threonine residues

**Fig. 2:**
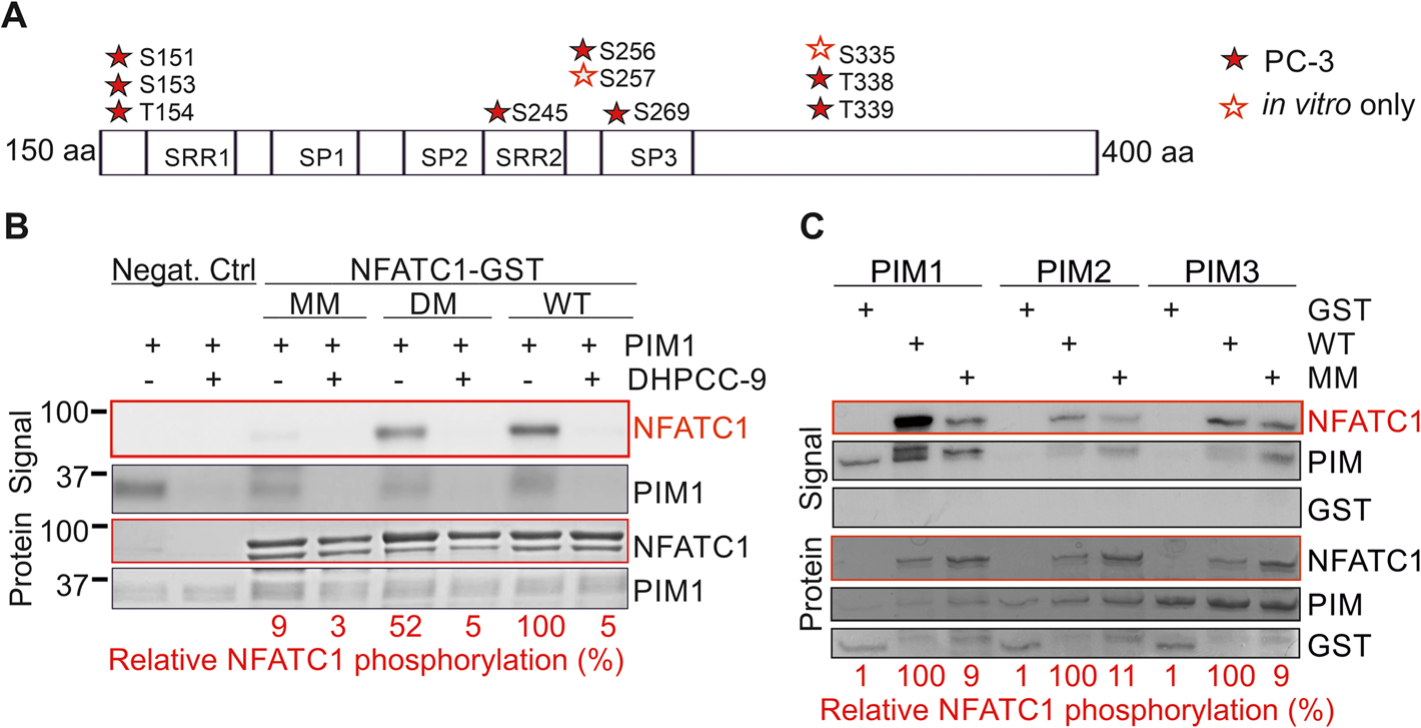
PIM1 phosphorylates NFATC1 at several novel target sites. **a** A schematic representation of the phosphorylation target sites for PIM1 in NFATC1 that were detected in vivo in PC-3 cells (marked with red filled stars) or only in vitro (marked with open stars), and that were mutated in this study. **b** Wild-type (WT) NFATC1 was mutated at two in vivo sites (S245A and S269A in the double mutant, DM) or at all detected sites (multi mutant, MM), grown in bacteria as GST fusion proteins and subjected to radioactive in vitro kinase assays with human PIM1 pretreated with DMSO (−) or 10 μM DHPCC-9 (+). Shown in the upper panel are the signal intensities of phosphorylated proteins (NFATC1 phosphorylation lined red), in the lower panel the total amounts of proteins (NFAT total protein loadings lined red), and under the panels the relative levels of phosphorylation of WT NFATC1 (100%) versus those of the mutants. **c** Similar kinase assays were performed also with human PIM2 and mouse PIM3

As we had previously shown that the PIM1 kinase phosphorylates NFATC1 and enhances its transcriptional activity [12], we now wanted to identify the as yet unknown PIM1 target sites in NFATC1 and to investigate their physiological roles in more detail. For this purpose, we carried out in vitro kinase assays with GST-tagged PIM-1 and NFATC1 (amino acids 1–418) produced in bacteria, and cell-based assays with YFP-tagged NFATC1 protein overexpressed in PC-3 cells. When phosphorylated NFATC1-derived peptides were subjected to mass spectrometry analyses, several novel phosphorylation target sites were discovered both from the in vitro samples and from the PC-3 cell-derived samples (Fig. 2a, Additional file 1: Table S3) in addition to those in vivo sites that we had already previously identified from COS-7 cells [27]. However, since more endogenously phosphorylated cellular sites were discovered than in vitro target sites for PIM1, it was evident that many of the in vivo sites were targeted by other kinases.

To be able to evaluate the functional impact of phosphorylation at putative PIM1 target sites, we mutated multiple serine or threonine residues in NFATC1 into alanines to create phosphodeficient mutants (Fig. 2a, Table 1, Additional file 1 Table S3). The mutated sites were primarily chosen based on the presence of PIM1 consensus target sequences [34] with basic residues preceding the in vivo phosphosites observed in PC-3 cells. Those were supplemented with close-by sites that had been phosphorylated by PIM1 in vitro. Stepwise mutagenesis resulted in approximately 50% (double mutant, DM) or 90% (multi mutant, MM) reduction in the ability of PIM1 to phosphorylate NFATC1 in vitro, while the previously validated PIM-selective inhibitor DHPCC-9 [4, 26] fully abrogated PIM1 autophosphorylation and PIM1-mediated NFATC1 phosphorylation (Fig. 2b). In addition to PIM1, also PIM2 and PIM3 were able to phosphorylate WT, but not MM NFATC1 in vitro (Fig. 2c).

### Both wild-type and phosphomutant NFATC1 interact with PIM1 in PC-3 cells

**Fig. 3:**
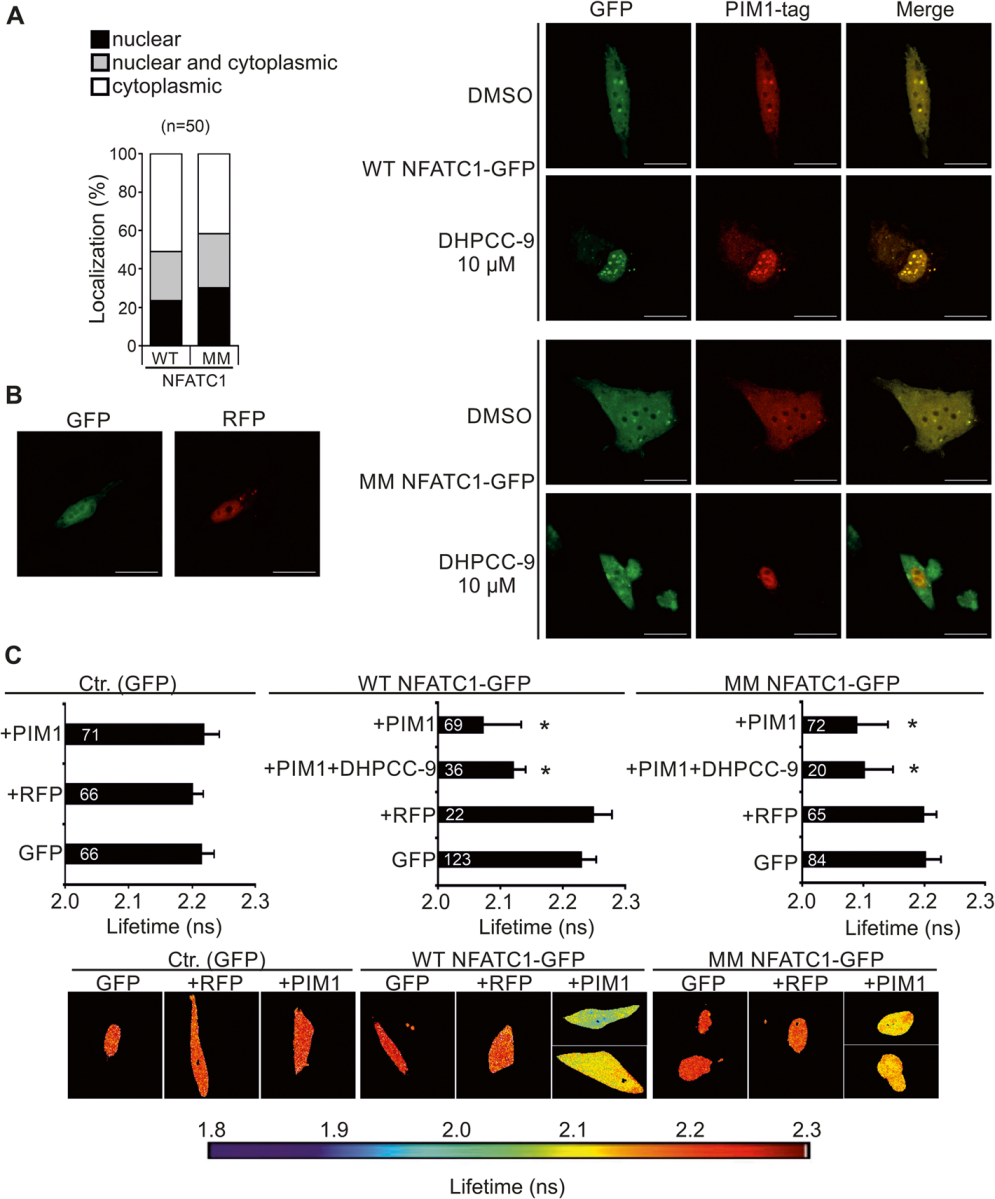
PIM1 interacts with NFATC1 in PC-3 cells. **a** Wild-type (WT) or multi mutant (MM) NFATC1 were transiently expressed in PC-3 cells and their subcellular localization patterns were analysed by confocal microscopy after staining with anti-Flag antibody. Shown are means from one experiment with three parallel samples. **b** The physical interactions between RFP-tagged PIM1 and GFP-tagged WT or MM NFATC1 proteins were analysed by fluorescence-lifetime imaging microscopy (FLIM) from samples of transiently transfected PC-3 cells. 24 h after transfection, cells were treated overnight with DMSO or 10 μM DHPCC-9. Shown on the left are representative images of negative control cells with expression of empty GFP or RFP vectors, while on the right are single channel or merged images of cells co-transfected with GFP- or RFP-tagged vectors. Scale bar 20 μm. **c** Shown are average GFP lifetimes from two independent FLIM experiments along with sample numbers inside the black bars as well as representative images with a heatmap. Lowest negative control (GFP + RFP) value was set as the limit for physical interaction

To assess the subcellular localization of WT versus phosphodeficient NFATC1, we transiently expressed them in PC-3 cells, where they showed similar localization patterns in the nucleus, in the cytoplasm or in both (Fig. 3a, Additional file 1: Figure S1C). As we had previously shown that NFATC1 and PIM1 can be co-immunoprecipitated with each other [12], we now wanted to determine whether the mutations in the PIM1 target sites affected either the colocalization or the physical interaction of GFP-tagged NFATC1 proteins with RFP-tagged PIM1. For these purposes, confocal microscopy and the fluorescence-lifetime imaging method (FLIM) were used as in our previous studies [23]. Both WT and MM NFATC1 showed nuclear co-localization (Fig. 3b) as well as interaction (Fig. 3c) with PIM1, as was evident from merged confocal images and from reduced lifetimes of GFP signals, respectively. Furthermore, the PIM inhibitor DHPCC-9 did not have major effects on the localizations or interactions, indicating that PIM-induced phosphorylation was not required there (Fig. 3b, c).

### Phosphorylation by PIM1 promotes NFATC1 activity

**Fig. 4:**
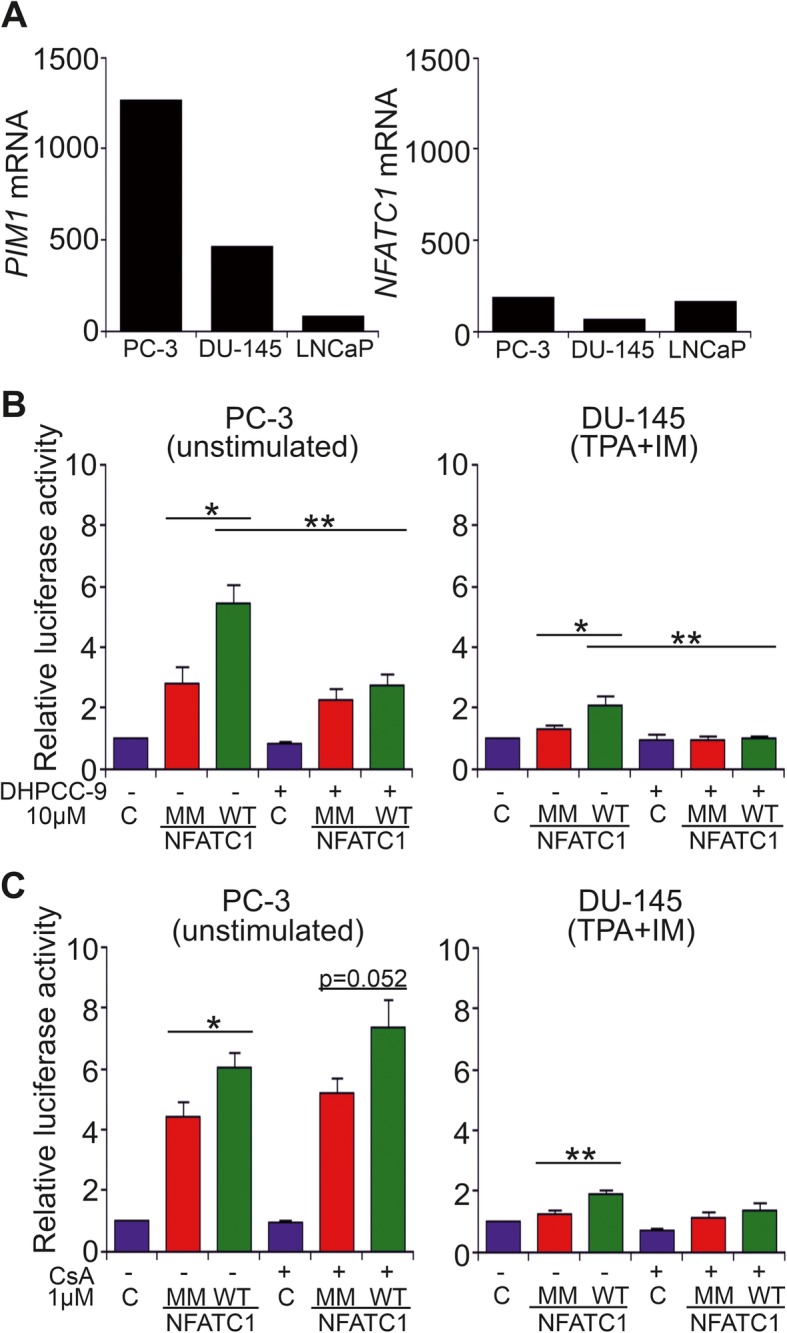
Effects of PIM-dependent phosphorylation on NFAT activity. **a** The expression levels of *PIM1* and *NFATC1* mRNAs were determined from our previously published RNA-sequencing dataset from PC-3, DU-145 and LNCaP cell lines [36]. **b** The impact of PIM-dependent phosphorylation on NFAT activity was analysed by luciferase assays in PC-3 and DU-145 cells that transiently expressed wild-type (WT) or multi mutant (MM) NFATC1. Cells were treated with either DMSO (−) or 10 μM DHPCC-9 (+). In addition, DU-145 cells had been pre-treated with TPA and IM. Shown are means of relative luciferase activities from two independent experiments with four parallel samples, the results of which had been normalized against the mock-transfected control samples. **c** Similar luciferase assays were performed also with cells treated with either EtOH (−) or 1 μM CsA (+)

To determine whether phosphorylation affects transcriptional activity of NFATC1, we transiently overexpressed WT and mutant NFATC1 proteins in three different prostate cancer cell lines, PC-3, DU-145 and LNCaP cells. Both PC-3 and DU-145 cells represent hormone-insensitive tumors, while LNCaP cells are hormone-sensitive, but carry mutated androgen receptors [35]. Based on our previously published RNA-sequencing dataset [36], endogenous *PIM1* mRNA expression levels were relatively high in PC-3 cells, lower in DU-145 cells and lowest in LNCaP cells, while relatively low *NFATC1* mRNA levels were observed for all cell lines (Fig. 4a).

According to data from NFAT-luciferase assays, PC-3 cells had clearly higher basal NFAT activity than DU-145 or LNCaP cells, although in DU-145 cells the activity could be increased by stimulation with TPA and the calcium ionophore ionomycin (Fig. 4b, Additional file 1: Figure S4A). This suggests that in contrast to PC-3 cells, NFAT nuclear translocation and activation are normally regulated by calcium and calcineurin in DU-145 cells. This conclusion was further supported by the ability of cyclosporin to slightly suppress NFAT activity in stimulated DU-145 cells, but not in any other cell samples (Fig. 4c, Additional file 1: Figure S4B).

The presence of overexpressed WT NFATC1 strongly enhanced NFAT activity in all three cell lines, while mutations in the PIM1 target sites or treatment of cells with the PIM inhibitor DHPCC-9 resulted in significantly compromised NFAT activities in PC-3 and DU-145 cells, but not in LNCaP cells (Fig. 4b, Additional file 1: Figure S4). These results indicated that full NFAT activity was dependent on phosphorylation of PIM target sites in PC-3 and DU145 cells, but PIM-independent in LNCaP cells, where nearly negligible *PIM* mRNA levels had been observed (Fig. 4a).

### Prostate cancer cell motility is regulated by NFATC1 phosphorylation

**Fig. 5:**
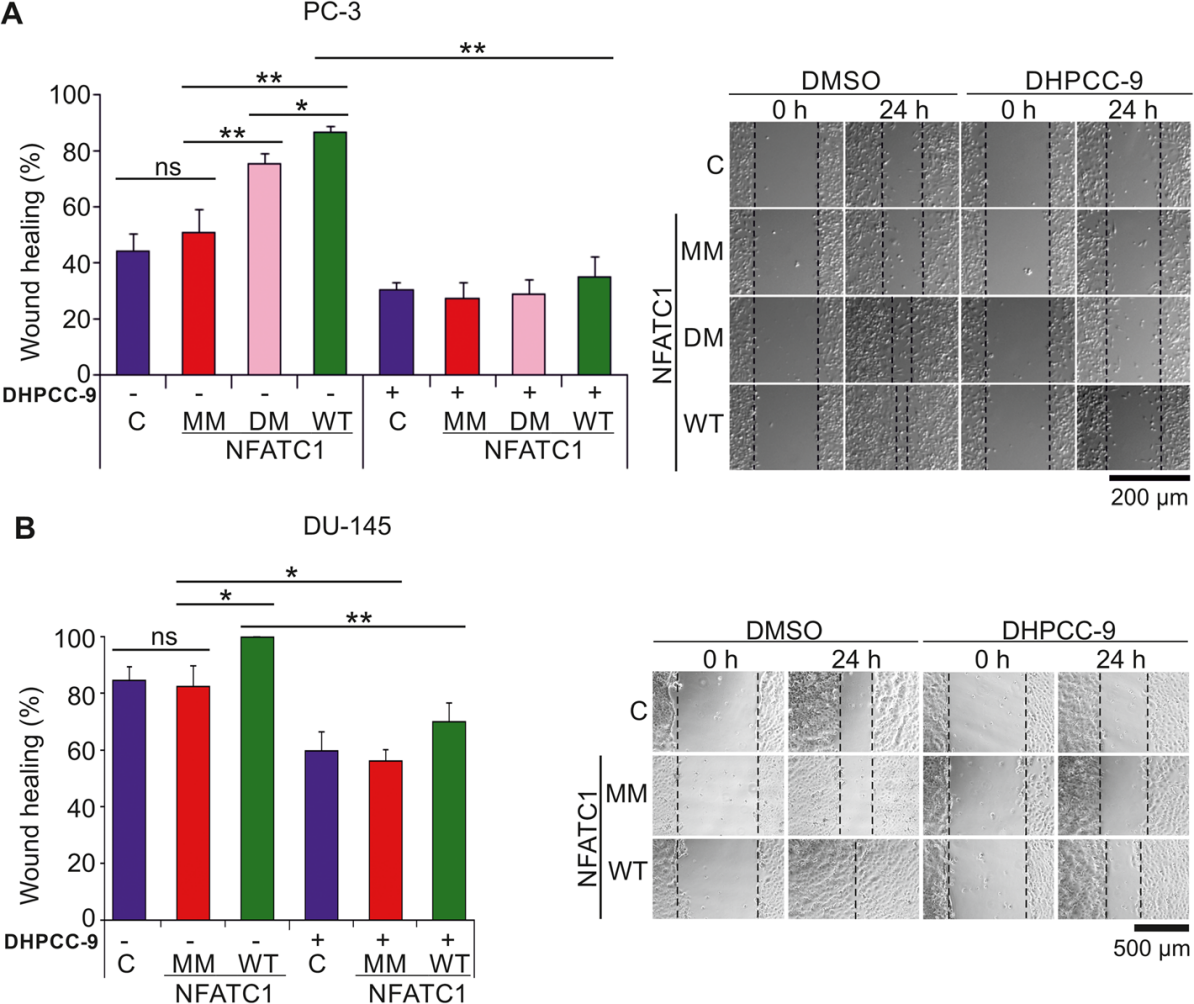
Lack of PIM1 target sites reduces the ability of NFATC1 to promote migration of prostate cancer cells. Wild-type (WT), double mutant (DM) or multi mutant (MM) NFATC1 were transiently expressed in PC-3 cells (**a**) or DU-145 cells (**b**). For wound healing assays, cell layers were scratched 24 h after transfection with a 10 μl pipette tip and the wounded areas were allowed to recover for another 24 h in the presence of either DMSO or 10 μM DHPCC-9. Shown are representative pictures taken at 0 h and 24 h time-points, and average wound healing percentages

As we had previously shown that PIM inhibition blocks the pro-migratory effects of NFATC1 in PC-3 cells [4], we wanted to investigate the role of NFATC1 phosphorylation in this context. In wound healing assays with PC-3 cells transiently overexpressing WT NFATC1 or phosphomutants, mutations in the PIM target sites significantly reduced the ability of NFATC1 to promote cell migration (Fig. 5a). While there were minor effects by the DM mutant and more pronounced effects by the MM mutant, PIM inhibition by DHPCC-9 completely blocked cell migration in each case. Similar wound healing experiments were also performed with DU-145 prostate cancer cells, which transiently overexpressed WT NFATC1 or the multi mutant. As in PC-3 cells, mutations in the PIM target sites abolished the ability of NFATC1 to promote cell migration (Fig. 5b). Also DHPCC-9 diminished motility, but less efficiently than in PC-3 cells, which migrated slightly slower than DU-145 cells.

Western blotting was used to confirm equivalent protein levels of NFATC1 and PIM family members in PC-3 cells (Additional file 1: Figure S3A). DHPCC-9 slightly reduced them, but did not significantly affect cell viability (Additional file 1: Figure S3A, B). Similar viability and protein expression data were obtained also from DU-145 cells (Additional file 1: Figure S3C). Additional wound healing assays were performed in PC-3 cells in the presence of mitomycin C to exclude effects of cell proliferation on cell migration (Additional file 1: Figure S3D), but no major differences were observed as compared to its absence (Fig. 5a). More interestingly, the triple mutant (TM) NFATC1 with intact S245 and S269 sites blocked cell migration almost as efficiently as MM lacking them, suggesting that the pro-migratory effects of NFATC1 were more dependent on phosphorylation of other PIM1 target sites.

**Fig. 6:**
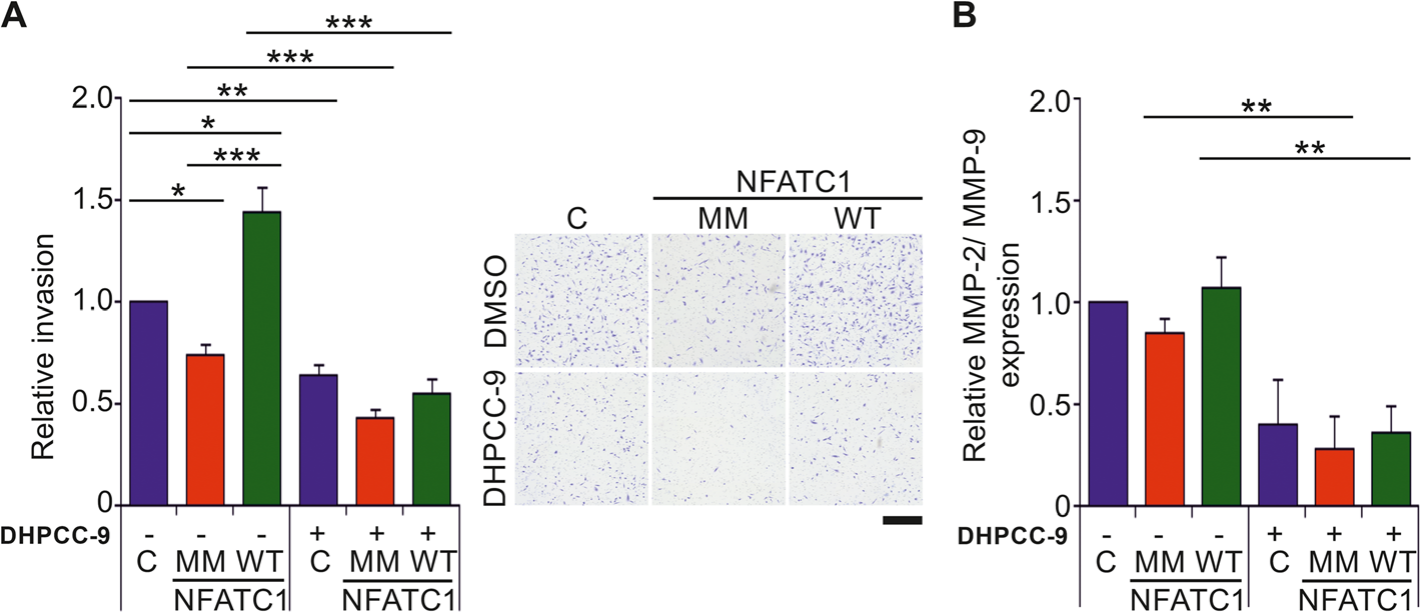
Lack of PIM1 target sites reduces also the ability of NFATC1 to enhance invasiveness of prostate cancer cells. **a** For invasion assays, PC-3 cells were grown in Boyden chambers in the absence (−) or presence (+) of 10 μM DHPCC-9. After 48 h, cells that had invaded through the membranes were fixed, stained with crystal violet and counted. Shown are relative invasion rates from two separate experiments with triplicate samples, the results of which had been normalized against the mock-transfected control samples. Shown are also representative pictures of the effects of wild-type (WT) or multi mutant (MM) NFATC1 on cell invasion after 48 h. Scale bar 500 μm. **b** Matrix metalloprotease (MMP) expression levels were measured by gelatinase activity assays from invasion sample media. Shown are relative MMP-2/MMP-9 expression levels from two separate experiments with three parallel samples

To investigate the role of NFATC1 phosphorylation in cell invasion, we carried out matrigel-based Boyden chamber invasion assays. There WT NFATC1 increased invasion of transiently transfected PC-3 cells through the membranes, while mutations in PIM targets sites in MM NFATC1 or the presence of the PIM inhibitor DHPCC-9 decreased it (Fig. 6a). NFATC1 protein levels were also monitored by western blotting in the invasion experiments (Additional file 1: Figure S3E). No major differences were observed in cell viability, except for a slight increase by MM NFATC1 at the later 72 h time-point (Additional file 1: Figure S3F). As activities of matrix metalloproteinases (MMPs), such as MMP-2 and MMP-9 are needed for cell invasion and may be regulated in an NFAT-dependent fashion also in our cells of interest [2], we analysed the effects of NFATC1 phosphorylation on their expression by gelatinase activity assays. The relative MMP expression levels were slightly, although not significantly reduced by MM NFATC1, while the decrease was more prominent with the PIM inhibitor DHPCC-9 (Fig. 6b). In each case, the MMP enzymatic activities correlated well with data from the invasion assays, suggesting that MMPs are relevant NFATC1 targets, whose activities can be indirectly regulated by PIM kinases.

### *ITGA5* is a putative target for phosphorylated NFATC1

To identify additional targets for the interplay between PIM1 and NFATC1, we designed microarray experiments, where we compared mRNA transcriptomes of PC-3 cells with or without stable overexpression of PIM1, and with or without transient overexpression of either WT or MM NFATC1. Real-time qPCR was first used to confirm overexpression of PIM1 and/or NFATC1 genes in the cell samples (Additional file 1: Figure S4A-B). With the microarrays, we performed three different types of comparisons: First, we compared parental PC-3 cells to their derivatives that stably expressed PIM1, to identify the genes that are up- or downregulated by elevated PIM1 expression. Secondly, we compared the PC-3 cells that had transiently been transfected with WT or MM NFATC1, to find genes that are controlled by the levels of NFATC1 activity. Finally, we compared PC-3 cells that both stably overexpressed PIM1 and transiently expressed WT or MM NFATC1, to unravel the genes regulated by PIM1-dependent phosphorylation of NFATC1. Genes with altered expression profiles in these three comparisons are listed in Additional file 1: Table S4.

**Fig. 7:**
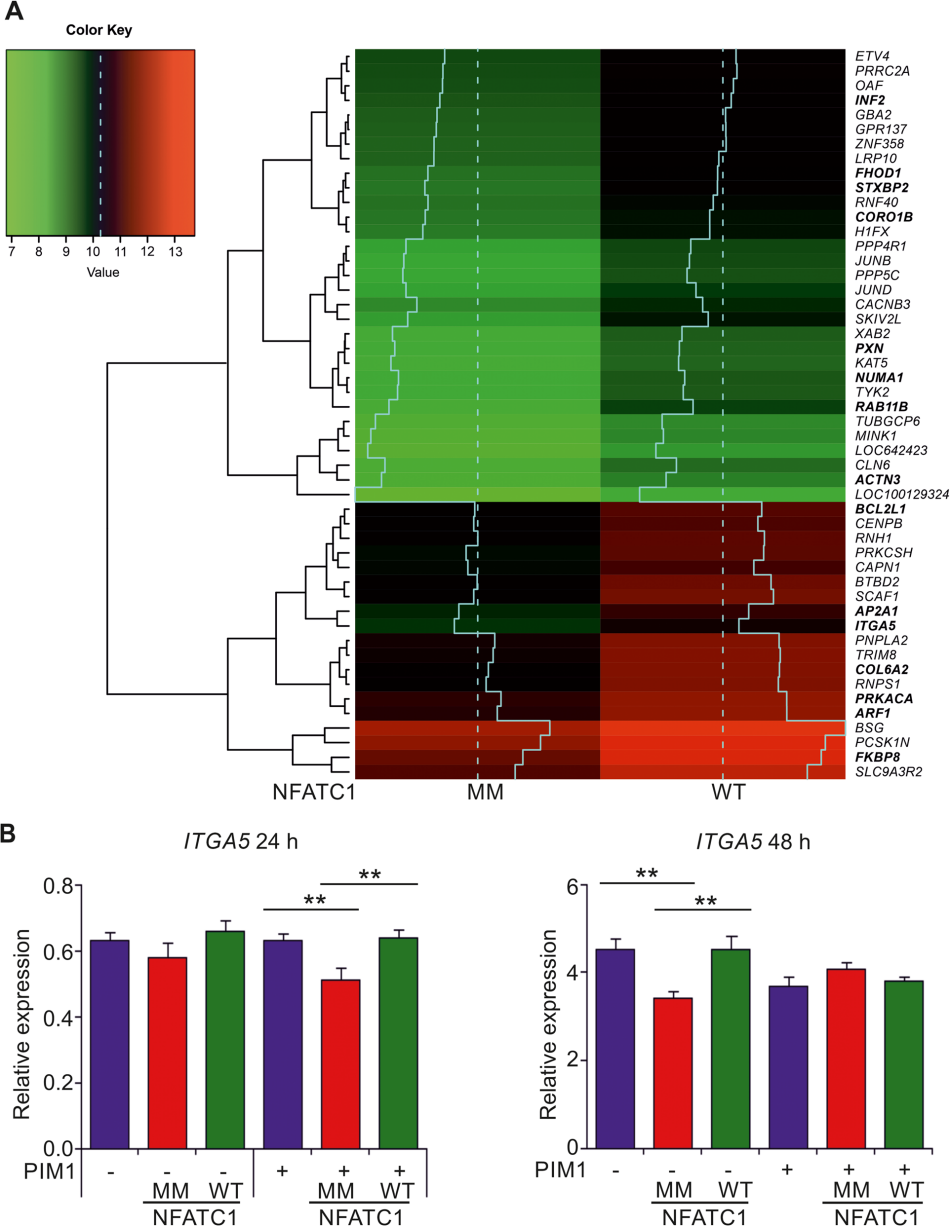
Microarray analysis reveals *ITGA5* as a putative PIM1/NFATC1 target gene. **a** Heatmap of the potential PIM1/NFATC1 target genes found from microarray analysis. Shown are fifty genes with highest log2 fold changes (logFC ≥1 and *P*-value ≤0,05), when PC-3 cells expressing PIM1 plus multi mutant (MM) NFATC1 were compared to cells expressing PIM1 plus wild-type (WT) NFATC1. Dashed line indicates the median of the expression values and solid line shows the expression levels more precisely in a diagrammatic form. Genes listed in bold are reviewed in more detail in the discussion. **b** Relative expression levels of *ITGA5* mRNA were analysed by real-time qPCR from microarray samples (right panel) and from another independent data set (left panel) after transient transfections of WT or MM NFATC1 to PC-3 cells without (−) or with (+) stable PIM1 overexpression. The data were normalized against *TBP* expression levels

Clustering analyses revealed that the cells overexpressing PIM1 and WT NFATC1 have a different profile as compared to the other samples (Additional file 1: Figure S4C). All the genes listed in Fig. 7a and Additional file 1: Table S4 showed higher mRNA levels in cells with WT NFATC1 than with MM, and their levels were lower also in the other control samples (Additional file 1: Figure S4C). Based on the observed gene expression profiles, we performed a canonical pathway analysis to determine, which cellular functions are primarily affected by the PIM-NFATC1 axis. We discovered five pathways that had significantly been enriched, many of which regulate cell adhesion and motility-related functions, like integrin, paxillin and FAK-signaling pathways (Additional file 2: Figure S6).

To validate the microarray data, we selected integrin alpha 5 (*ITGA5*) for more detailed expression analysis, as it is involved in the regulation of cell adhesion to matrices such as fibronectin [37, 38], and as we have previously connected PIM inhibition to decreased adhesion to fibronectin [39]. When we compared the expression levels of *ITGA5* mRNA between one independent data set (24 h after transfections) with microarray samples (48 h after transfections), we observed decreased expression in cells with MM NFATC1 as compared to control cells or cells with WT NFATC1 (Fig. 7b). These differences resembled those observed in MMP assays (Fig. 6b), and were statistically significant after 24 h in the cells stably overexpressing PIM1 and after 48 h in the control cell line, suggesting a role for PIM1-mediated phosphorylation and activation of NFATC1 in regulating *ITGA5* mRNA expression levels.

Our data prompted us to examine clinical prostate cancer samples for their expression levels of *PIM1*, *NFATC1* and/or *ITGA5* mRNAs. Therefore, we performed pairwise comparisons of these three genes in three independent prostate cancer patient-derived datasets [31–33]. The expression levels of *PIM1* and *ITGA5* or *NFATC1* and *ITGA5* mRNAs positively correlated in all datasets (Additional file 2: Figure S5). Most interestingly, the positive correlation between *NFATC1* and *ITGA5* increased along the Gleason score, with the strongest correlation in prostate cancer patients with Gleason ≥8.

## Discussion

Here we have analysed the functional interactions of PIM and NFATC1 proteins in several prostate cancer cell lines. We have identified multiple PIM target sites in NFATC1 that are phosphorylated in vitro and/or in cells, and are essential for the transcriptional activity of NFATC1 as well as for its pro-migratory and pro-invasive effects. By contrast, the physical interactions or colocalization of PIM1 and NFATC1 are not affected by PIM-dependent phosphorylation. In addition to PIM1, also PIM2 and PIM3 can phosphorylate NFATC1, adding it to the growing list of substrates shared by all PIM family members [16].

While our study was in progress, additional kinases targeting NFATC1 were identified. Phosphorylation by the IkB kinase epsilon (IKKε) was shown to inhibit NFATC1 activity [40], whereas phosphorylation by the DYRK1A kinase increased NFATC1 protein stability by interfering with NFATC1 ubiquitination and degradation [41]. We identified two IKKε target sites (Ser151 and Ser161) and one DYRK1A site (Ser278) as cellular phosphorylation sites of NFATC1 in PC-3 cells, but our multi mutant NFATC1 protein lacked only one of them (Ser 151), suggesting that the effects of the mutant were mostly due to lack of PIM-dependent phosphorylation. This conclusion was further supported by our observations that the double mutant lacking known PKA target sites (Ser245 and Ser269; 11) promotes cell migration nearly as efficiently as wild-type NFATC1, while the triple mutant with intact PKA target sites inhibits cell motility almost as much as the multi mutant.

In this study, we have shown that the PC-3 prostate cancer cells exhibit constitutive NFAT activity. This is in contrast to most cells, where upstream activation of the calcium- and calcineurin-dependent pathway is required to allow NFAT family members to enter the nucleus and stimulate transcription there [1, 2]. This may not be a general feature of prostate cancer cells, since in another hormone-insensitive cell line, DU-145, NFAT activity could be enhanced by the calcium ionophore ionomycin and inhibited by the calcineurin inhibitor cyclosporin A. Yet in both cell lines, the transcriptional as well as pro-migratory activities of NFATC1 were similarly compromised by mutations in the PIM target sites. As the PIM-selective inhibitor DHPCC-9 blocked the activities of NFATC1 even more efficiently, this suggests that it affects additional downstream targets, only some of which are shared by PIM1 and NFATC1.

In our microarray analyses of transfected PC-3 cell samples, we were able to identify novel putative PIM1/NFATC1 target genes, which were more abundantly expressed in the presence of both PIM1 and wild-type NFATC1, but less in cells expressing the multi mutant NFATC1 or in other types of control cells. Thus, expression of all these target genes may be upregulated by PIM1-dependent phosphorylation of NFATC1. The putative PIM1/NFATC1 target genes included one encoding for the known PIM substrate NUMA1 (nuclear mitotic apparatus protein 1 [42];). Otherwise the target genes could be divided into several groups based on the types of proteins encoded by them, including regulators of transcription, cell cycle, cell survival, cell motility, cell adhesion as well as intracellular trafficking. As expected, there were several genes involved in the NFAT signaling pathway [43], such as those encoding the catalytic subunit alpha of protein kinase A (PRKACA) and the FK506 immunosuppressant-binding immunophilin protein FKBP8. The latter protein also acts as a chaperone for the anti-apoptotic BCL2 protein, the expression levels of which have previously been shown to be upregulated by PIM kinases [44]. In addition, the BCL2 homolog BCL2L1 was listed there as also several genes encoding proteins involved in intracellular trafficking (RAB11B, STXBP2, AP2A1, ARF1). Maybe most interestingly in regard to our data on promotion of prostate cancer cell motility by PIM1 and NFATC1, there were several genes encoding regulators of the cytoskeletal actin network (INF2, FHOD1, ACTN3, CORO1B) and cell adhesion (COL6A2, PXN, ITGA5).

As signaling pathways involving integrins were highly enriched in our canonical pathway analysis, we picked ITGA5 for further expression analyses. Integrins are well-known cellular adhesion receptors that connect cells to the extracellular matrix and have been implicated in multiple steps of tumorigenesis [45]. ITGA5 has an essential role in cell adhesion, migration and tumor invasion [46–48]. Interestingly, previous experiments have linked both PIM and NFAT family members to integrin-mediated cell adhesion or motility. NFATC1 binds to the *ITGB3* promoter in osteoclast precursor cells, while NFATC2 and NFAT5 promote ITGA6/ITGB4-mediated cell invasion in breast cancer [49, 50]. Furthermore, PIM inhibition decreases cell adhesion to collagen and fibronectin matrices via different integrin subunits [38]. While no clear PIM-dependent changes in integrin activity or expression have previously been reported, we now found correlations between *PIM1* or *NFATC1* mRNA expression levels with *ITGA5*, both in PC-3 cells and in prostate cancer patient-derived samples. However, more detailed studies are needed to determine how critical *ITGA5* or other genes identified by the microarray analyses are in mediating the pro-motility effects of PIM and/or NFATC1 proteins.

## Conclusions

In conclusion, we have shown that phosphorylation of PIM1 target sites stimulates the transcriptional activity of NFATC1 and enhances its ability to promote prostate cancer cell migration and invasion. Thereby, the interplay between PIM kinases and NFATC1 may also provide possibilities for therapeutic interventions against metastatic prostate cancer through combinatory approaches involving PIM-selective kinase inhibitors.

## Supplementary information

**Additional file 1: Table S1.** Primers for site-directed mutagenesis in NFATC1. **Table S2.** Primers for qRT-PCR. **Table S3.** Novel NFATC1 phosphorylation sites. **Table S4**. Phosphorylation-dependent differences in the expression of PIM/NFATC1 target genes in PC-3 cells.

**Additional file 2: Figure S1.** Lack of PIM target sites does not affect subcellular localization of NFATC1 A. **Figure S2.** Effects of PIM-dependent phosphorylation on NFAT activity. **Figure S3.** Lack of PIM1 target sites reduces the ability of NFATC1 to promote cancer cell motility. **Figure S4.** Microarray analysis reveals phosphorylation-dependent differences in the expression of PIM/NFATC1 target genes in PC-3 cells. **Figure S5.** Integrin signaling pathway is enriched in PIM1 and NFATC1 expressing cells. **Figure S6.** ITGA5 mRNA expression levels correlates with those of *PIM1* and *NFATC1* in clinical prostate cancer samples.

## Data Availability

The microarray data has been deposited to the Gene Expression Omnibus (GEO, National Center for Biotechnology Information, Bethesda, MD, USA) with series entry number GSE120133, and is available from there: https://www.ncbi.nlm.nih.gov/geo/query/acc.cgi?acc=GSE120133. Other experimental data sets used and analysed during the current study as well as materials prepared are available from the corresponding author on reasonable request.

## Abbreviations

ACTB: ß-actin
CsA: Cyclosporine A
DHPCC-9: Pan-PIM inhibitor
DM: double mutant
DN: dominant negative
IM: Ionomycin
ITGA5: Integrin alpha 5
LSB: Laemmli sample buffer
MM: multi mutant
MMP: Matrix metalloproteinase
mSRR: constitutively active mutant of NFATC1
NFAT: Nuclear factor of activated T cells
ns: non-significant difference
TBP: TATA-binding protein
TM: triple mutant
TPA: 12–0-tetradecanoyl-phorbol-13-asetate
WT: wild-type
