# Supplementary material for: Phosphorylation of NFATC1 at PIM1 target sites is essential for its ability to promote prostate cancer cell migration and invasion
Source: Cell Commun Signal. 2019 Nov 15;17:148. doi: 10.1186/s12964-019-0463-y (PMC6858710; doi:10.1186/s12964-019-0463-y)
Supplement: Supplementary file 1 — Additional file 1: Table S1. Primers for site-directed mutagenesis in NFATC1. Table S2. Primers for qRT-PCR. Table S3. Novel NFATC1 phosphorylation sites. Table S4. Phosphorylation-dependent differences in the expression of PIM/NFATC1 target genes in PC-3 cells. [file 12964_2019_463_MOESM1_ESM.docx]

**Additional file 1**

**Table S1.** Primers for site-directed mutagenesis in NFATC1**.** Listed are primer sequences used for designed mutations in PIM1 phosphorylation target sites of NFATC1. Nucleotides in bold were mutated and the restriction sites used to help in screening of the mutations are underlined. Original sequences are listed under the mutated sequences.

**Mutant primers 1.**

| **Ser245** Restriction site PvuII = CAG/CTG (32 nt) |
| --- |
| F : 5’ CCTCGCCCCGCGCA**GCT**GTCACTGAGGAGAGC 3’ |
| R : 5’ GCTCTCCTCAGTGACA**GCT**GCG**C**GGGGCGAGG 3’ |

Original sequence from first site:

| F: 5’ CCTCGCCCCGCGCA**AGC**GTCACTGAGGAGAGC 3’ |
| --- |
| R: 5’ GCTCTCCTCAGTGACA**AGC**GCGCGGGGCGAGG 3’ |

**Mutant primers 2.**

| **Ser269** Restriction site *MluI* = A/CGCGT (38 nt) |
| --- |
| F : 5’ GCAACAAGAGGAAGTAC**GCG**TTGAACGGCCGGCAGCCG 3’ |
| R : 5’ CGGCTGCCGGCCGTTCAAC**GCG**TACTTCCTCTTGTTGC 3’ |

Original sequence from first site:

| F : 5’ GCAACAAGAGGAAGTAC**AGC**TTGAACGGCCGGCAGCCG 3’ |
| --- |
| R: 5’ CGGCTGCCGGCCGTTCAAC**AGC**TACTTCCTCTTGTTGC 3’ |

**Mutant primers 3.**

| **Ser151 + Ser153 + Thr154** Restriction site *NotI* = GC/GGCCGC (41nt) |
| --- |
| F: 5’ CCTAGCTCCAAACGG**G**CCCCC**G**C**GG**C**C**GCCGCCACGCTGAG 3’ |
| R: 5’ CTCAGCGTGGCGGC**G**G**CC**G**C**GGGGG**C**CCGTTTGGAGCTAGG 3’ |

Original sequence from second site:

| F: 5’ CCTAGCTCCAAACGG**T**CCCCC**T**C**CA**C**G**GCCACCACGCTGAG 3’ |
| --- |
| R: 5’ CTCAGCGTGGTGGC**C**G**TG**G**A**GGGGG**A**CCGTTTGGAGCTAGG 3’ |

**Mutant primers 4.**

| **Ser256 + Ser257** Restriction site *NheI* = G/CTAGC (33 nt) |
| --- |
| F: 5’ CTGGGTGCCCGC**G**CC**G**CCAGACCCGC**TAG**CCC**G** 3’ |
| R: 5’ **C**GGG**CTA**GCGGGTCTGG**C**GG**C**GCGGGCACCCAG 3’ |

Original sequence from third site:

| F 5’ CTGGGTGCCCGC**T**CC**T**CCAGACCCGC**GTC**CCC**T** 3’ |
| --- |
| R 5’ **A**GGG**GAC**GCGGGTCTGG**A**GG**A**GCGGGCACCCAG 3’ |

**Mutant primers 5.**

| **Ser335 + Thr338 + Thr339** Restriction site *XhoI* =C/TCGAG (36 nt) |
| --- |
| F 5’ GTCCCTGTCAAG**G**CCCGCAAG**G**CC**G**CCCT**C**GAGCAG 3’ |
| R 5’ CTGCTC**G**AGGG**C**GG**C**CTTGCGGG**C**CTTGACAGGGAC 3’ |

Original sequence from fourth site:

| F 5’ GTCCCTGTCAAG**T**CCCGCAAG**A**CC**A**CCCT**G**GAGCAG 3’ |
| --- |
| R 5’ CTGCTC**C**AGGG**T**GG**T**CTTGCGGG**A**CTTGACAGGGAC 3’ |

**Table S2.** Primers for qRT-PCR. All the primer sequences (*TPB*, *PIM1*, *NFATC1* and *ITGA5*) used for qRT-PCR analysis.

***TPB* primers for qRT-PCR**

| F: 5’ GAATATAATCCCAAGCGGT 3’ |
| --- |
| R: 5’ ACTTCACATCACAGCTCCCC 3’ |

***PIM1* primers for qRT-PCR**

| F: 5’ CTGGGGAGAGCTGCCTAATG 3’ |
| --- |
| R: 5’ GCTCCCCTTTCCGTGATGAA 3’ |

***NFATC1* primers for qRT-PCR**

| F: 5’ AAGCACCAGCTTTCCAGTCC 3’ |
| --- |
| R: 5’ TGCATAGCCATAGTGTTCTTCC 3’ |

***ITGA5* primers for qRT-PCR**

| F: 5’ AGACTTCTTTGGCTCTGCCC 3’ |
| --- |
| R: 5’ ACATGGTTCTGCTCCCCAAA 3’ |

**Table S3.** Novel NFATC1 phosphorylation sites**.** The *in vivo* or *in vitro* PIM1 target sites in NFATC1 identified in this study have been separated from sites previously identified from COS-7 cells (27), from high-throughput (HT) analyses listed by PhosphoSitePlus® (phosphosite.org) or more specifically for other kinases, such as IKKε (40), PKA (10, 11) or DYRK1A (41). Note that the table does not include all possible phopshorylation sites of NFATC1. The mutated sites in double mutant (DM), triple mutant (TM) or multi mutant (MM) NFATC1 have been indicated with bold fonts.

| Amino acid residue | *In vivo* sites  in PC-3 cells | *In vivo* sites in COS-7 cells | *In vitro* site  for PIM1 | *In vitro* or *in vivo* sites  for other kinases | NFATC1 mutant |
| --- | --- | --- | --- | --- | --- |
| **S151** | ***x*** | *-* | *-* | IKKε | TM, MM |
| **S153** | **x** | - | - | - | TM, MM |
| **T154** | **x** | - | - | - | TM, MM |
| T156 | x | - | - | - | - |
| S158 | x | - | - | HT | - |
| S161 | x | - | - | IKKε | - |
| S175 | x | - | - | - | - |
| S176 | x | - | - | - | - |
| T178 | x | - | - | - | - |
| T179 | x | - | - | - | - |
| **S245** | **x** | **x** | **x** | PKA | DM, MM |
| **S256** | **x** | - | - | - | TM, MM |
| **S257** | **-** | - | **x** | HT | TM, MM |
| **S269** | **x** | - | **x** | PKA | DM, MM |
| S278 | x | x | - | DYRK1A | - |
| S282 | x | x | - | HT | - |
| T284 | x | x | - | - | - |
| S286 | x | x | - | - | - |
| S290 | x | x | - | HT | - |
| **S335** | - | - | **x** | - | TM, MM |
| **T338** | **x** | - | **x** | - | TM, MM |
| **T339** | **x** | - | **x** | - | TM, MM |
| T359 | x | x | - | HT | - |

**Table S4.** Phosphorylation-dependent differences in the expression of PIM/NFATC1 target genes in PC-3 cells. Top 50 genes that were differentially expressed in PC-3 cells overexpressing PIM1 and either wild-type (WT) or multi mutant (MM) NFATC1. In this table, relative gene expression changes when WT NFATC1 expressing samples were compared to MM NFATC1 expressing samples are shown. Thresholds to consider gene expression change significant, was logFC ≥1 and p-value ≤ 0,05.

| Gene acronym | logFC | p-value |
| --- | --- | --- |
| RAB11B | -1,66212 | 7,82E-06 |
| SKIV2L | -1,5609 | 0,008398 |
| SCAF1 | -1,53947 | 0,000207 |
| FKBP8 | -1,50098 | 2,32E-05 |
| PRKCSH | -1,47978 | 2,64E-07 |
| BSG | -1,42546 | 0,000724 |
| PRRC2A | -1,37796 | 2,12E-05 |
| BTBD2 | -1,37431 | 9,05E-08 |
| STXBP2 | -1,33762 | 0,00013 |
| MINK1 | -1,33609 | 0,00018 |
| RNPS1 | -1,32754 | 0,000738 |
| SLC9A3R2 | -1,32527 | 0,010213 |
| ZNF358 | -1,32407 | 1,29E-08 |
| CLN6 | -1,30607 | 0,000426 |
| INF2 | -1,30467 | 0,000231 |
| ETV4 | -1,30329 | 9,44E-05 |
| COL6A2 | -1,29559 | 4,37E-07 |
| OAF | -1,29146 | 7,9E-07 |
| JUND | -1,26384 | 2,41E-06 |
| AP2A1 | -1,26184 | 0,00019 |
| FHOD1 | -1,25799 | 0,010966 |
| GPR137 | -1,25276 | 1,25E-06 |
| XAB2 | -1,25134 | 1,69E-08 |
| ARF1 | -1,25123 | 9,47E-05 |
| GBA2 | -1,22427 | 0,003948 |
| LOC642423 | -1,21137 | 1,35E-06 |
| KAT5 | -1,19893 | 0,008641 |
| TRIM8 | -1,1843 | 0,003075 |
| BCL2L1 | -1,18013 | 5,27E-05 |
| H1FX | -1,17839 | 3,17E-07 |
| TUBGCP6 | -1,17141 | 0,000119 |
| RNH1 | -1,17026 | 7,49E-05 |
| NUMA1 | -1,15801 | 7,06E-08 |
| CAPN1 | -1,1518 | 0,000105 |
| CACNB3 | -1,14664 | 0,001315 |
| PRKACA | -1,14488 | 3,88E-07 |
| TYK2 | -1,14358 | 1,33E-07 |
| JUNB | -1,141 | 2,52E-06 |
| RNF40 | -1,13097 | 6,91E-07 |
| LRP10 | -1,12263 | 0,000739 |
| PCSK1N | -1,11936 | 0,000342 |
| PPP4R1 | -1,1186 | 0,007407 |
| PXN | -1,11312 | 1,06E-08 |
| ITGA5 | -1,11235 | 1,32E-06 |
| ACTN3 | -1,11218 | 0,000303 |
| CORO1B | -1,10961 | 2,97E-08 |
| LOC100129324 | -1,1086 | 0,00229 |
| PNPLA2 | -1,10583 | 5,15E-06 |
| PPP5C | -1,10104 | 1,05E-08 |
| CENPB | -1,10039 | 0,001016 |
