# Supplementary material for: Phosphorylation of NFATC1 at PIM1 target sites is essential for its ability to promote prostate cancer cell migration and invasion
Source: Cell Commun Signal. 2019 Nov 15;17:148. doi: 10.1186/s12964-019-0463-y (PMC6858710; doi:10.1186/s12964-019-0463-y)
Supplement: Supplementary file 2 — Additional file 2: Figure S1. Lack of PIM target sites does not affect subcellular localization of NFATC1 A. Figure S2. Effects of PIM-dependent phosphorylation on NFAT activity. Figure S3. Lack of PIM1 target sites reduces the ability of NFATC1 to promote cancer cell motility. Figure S4. Microarray analysis reveals phosphorylation-dependent differences in the expression of PIM/NFATC1 target genes in PC-3 cells. Figure S5. Integrin signaling pathway is enriched in PIM1 and NFATC1 expressing cells. Figure S6. ITGA5 mRNA expression levels correlates with those of PIM1 and NFATC1 in clinical prostate cancer samples. [file 12964_2019_463_MOESM2_ESM.docx]

**
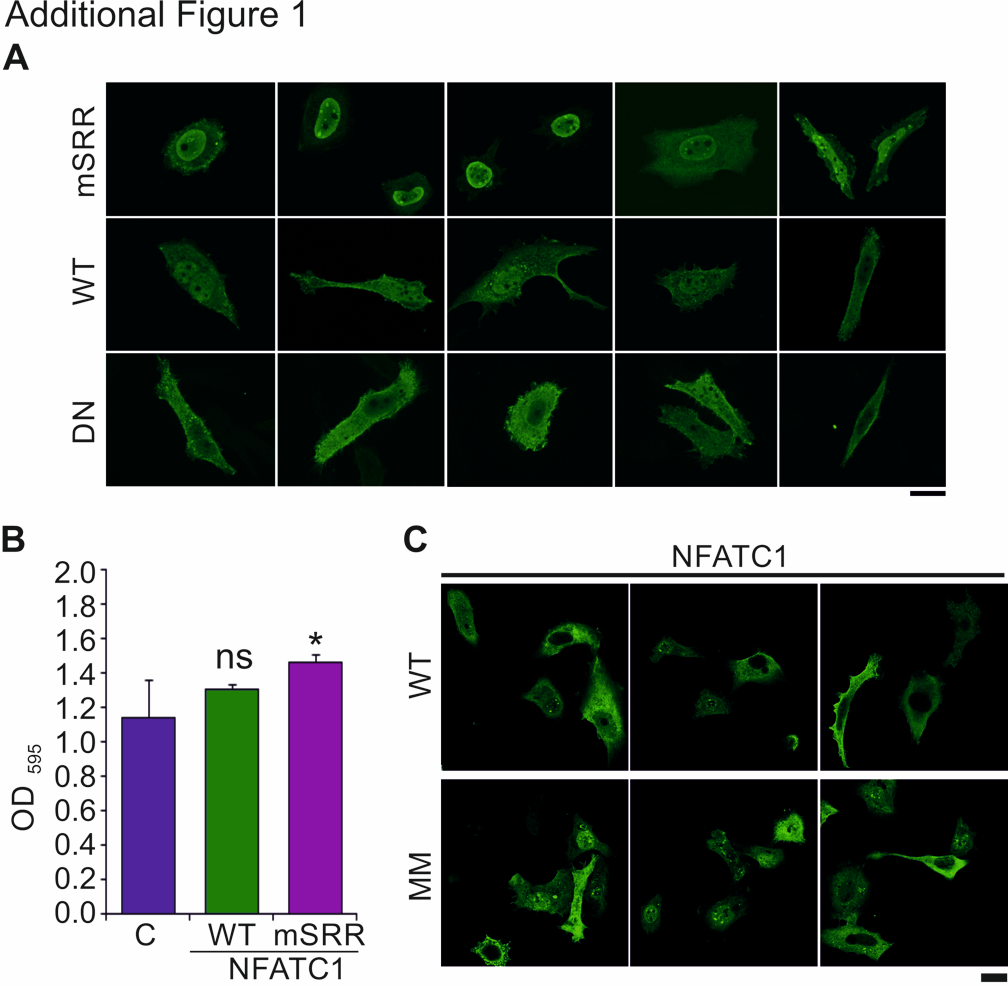
**

**Figure S1** Lack of PIM target sites does not affect subcellular localization of NFATC1. **a** Representative confocal microscopy pictures for data shown in Figure 1d on the subcellular localization patterns of transiently expressed wild-type (WT) NFATC1, the constitutively active (mSRR) mutant and the dominant negative (DN) mutant. **b** The average effects of WT NFATC1 or the mSRR mutant on cell viability were analysed by the MTT assay from three parallel samples used for Figure 1E. **c** Representative confocal microscopy pictures for data shown in Figure 3a on the subcellular localization patterns of transiently expressed WT or multi mutant (MM) NFATC1. Scale bars 20 µm.





**Figure S2** Effects of PIM-dependent phosphorylation on NFAT activity**.** Original NFAT-dependent luciferase activities for relative data partially shown in Figure 4b and C from PC-3, DU-145 and LNCaP cell lines transiently transfected with wild-type (WT) or multi mutant (MM) NFATC1. Part of DU-145 cells were pre-treated with TPA and IM, after which all cells were treated with either DMSO (-) or DHPCC-9 (+) (**a**), or with ETOH (-) or CsA (+) (**b**).





**Figure S3** Lack of PIM1 target sites reduces the ability of NFATC1 to promote cancer cell motility. **a** Western blotting was used to measure levels of endogenously expressed PIM family members and ectopically expressed Flag-tagged NFATC1 proteins from the experiment for Figure 5a. **b** The average relative effects of wild-type (WT), double mutant (DM) or multi mutant (MM) NFATC1 on cell viability were analysed by the MTT assay from three parallel samples used for Figure 5a. The data were normalized against mock-transfected control (C) cells. **c** The average relative effects of WT or MM NFATC1 on DU-145 cell viability were analysed by the AlamarBlue® viability assay from three parallel samples used for Figure 5b. They were normalized against values at the starting point, and the NFATC1 protein expression levels detected by Western blotting from the same experiment. β Tubulin staining was used as a loading control. **d-e** Wound healing assays were performed in PC-3 cells also in the presence of mitomycin C to exclude effects of cell proliferation. A triple mutant (TM) of NFATC1 was included in addition to other mutants. Shown are average wound healing percentages from representative experiments with three parallel samples (**d**), and the NFATC1 protein expression levels detected by Western blotting from the same experiment (**e**). Fibrillarin staining was used as a loading control. **f** The average relative effects of WT or MM NFATC1 on cell viability were analysed by the AlamarBlue® viability assay at three time-points (0 h, 24 h and 72 h after transfection) from PC-3 cells used in invasion assays in Figure 6a, and the data were normalized against values at the starting point.

**

**

**Figure S4** Microarray analysis reveals phosphorylation-dependent differences in the expression of PIM/NFATC1 target genes in PC-3 cells. Relative expression levels of *PIM1* (**a**) or *NFATC1* (**b**) mRNAs from microarray samples, as analysed by real-time qPCR and normalized against *TBP* levels. Expression levels were measured from PC-3 cell derivatives with (+) or without (-) stable PIM1 overexpression, and with transient overexpression of wild-type (WT) or multi mutant (MM) NFATC1. **c** Heatmap of the 50 genes with highest log2 fold changes (logFC >1 and P-value ≤ 0,05) observed in all the three array comparisons. All genes in bold are reviewed more in detail in discussion.

**

**

**Figure S5** Integrin signaling pathway is enriched in PIM1 and NFATC1 expressing cells. IPA (Ingenuity Pathway Analysis, Ingenuity Systems) was used for functional enrichment and detection of pathways with significant alterations based on microarray gene expressions. In canonical pathway analysis -log(p-values) over threshold 2.5 were considered significant.

**

**

**Figure S6** *ITGA5* mRNA expression levels correlates with those of *PIM1* and *NFATC1* in clinical prostate cancer samples**.** *ITGA5* mRNA expression levels were compared with *PIM1* or *NFATC1* mRNA levels by using three datasets of human prostate carcinomas: **a-b** Tampere PC sequencing data (Tre PCa; 33), **c-d** Integrative Genomic Profiling of Human Prostate Cancer microarray data (PCa; 32) or **e-i** The Cancer Genome Atlas (TCGA) - Prostate adenocarcinoma RNA-Sequencing data (31).
